# B cells reappear less mature and more activated after their anti-CD20–mediated depletion in multiple sclerosis

**DOI:** 10.1073/pnas.2012249117

**Published:** 2020-09-30

**Authors:** Nitzan Nissimov, Zivar Hajiyeva, Sebastian Torke, Katja Grondey, Wolfgang Brück, Silke Häusser-Kinzel, Martin S. Weber

**Affiliations:** ^a^Institute of Neuropathology, University Medical Center, 37075 Göttingen, Germany;; ^b^Department of Neurology, University Medical Center, 37075 Göttingen, Germany

**Keywords:** multiple sclerosis, anti-CD20 therapy, B cells, T cells, repletion

## Abstract

B cell depletion via anti-CD20 antibodies is a highly effective treatment for multiple sclerosis (MS). However, little is known about the maturation/activation stage of the returning B cell population after treatment cessation and the wider effects on other immune cells. In the present study, 15 relapsing-remitting MS patients receiving 1,000 mg of rituximab were included. B, T, and myeloid cells were analyzed before anti-CD20 administration and in different time intervals thereafter over a period of 24 mo. In comparison to the phenotype before anti-CD20 treatment, the reappearing B cell pool revealed a less mature and more activated phenotype: 1) reappearing B cells were significantly enriched in transitional (before: 10.1 ± 1.9%, after: 58.8 ± 5.2%) and mature naive phenotypes (before: 45.5 ± 3.1%, after: 25.1 ± 3.5%); 2) the frequency of memory B cells was reduced (before: 36.7 ± 3.1%, after: 8.9 ± 1.7%); and 3) reappearing B cells showed an enhanced expression of activation markers CD25 (before: 2.1 ± 0.4%, after: 9.3 ± 2.1%) and CD69 (before: 5.9 ± 1.0%, after: 21.4 ± 3.0%), and expressed significantly higher levels of costimulatory CD40 and CD86. T cells showed 1) a persistent increase in naive (CD4^+^: before: 11.8 ± 1.3%, after: 18.4 ± 3.4%; CD8^+^: before: 12.5 ± 1.4%, after: 16.5 ± 2.3%) and 2) a decrease in terminally differentiated subsets (CD4^+^: before: 47.3 ± 3.2%, after: 34.4 ± 3.7%; CD8^+^: before: 53.7 ± 2.1%, after: 49.1 ± 2.7%).

## Significance

Anti-CD20–mediated B cell depletion is a highly effective therapy in MS. However, long-term effects of this approach on the immune system are not yet characterized in detail. After cessation of anti-CD20 treatment, B cells reappear immature yet highly activated. In addition, anti-CD20 treatment exerts long-lasting effects on T cells, which may be important for its clinical effect. These findings may help in guiding therapeutic decisions with regard to treatment intervals and follow-up therapies.

Multiple sclerosis (MS) is a chronic inflammatory demyelinating disease of the central nervous system. T cells have been considered the major effector cell type in MS, but nowadays other immune cells, such as B cells or myeloid cells appear to be equally important (1). This conceptual shift was substantiated by the success of B cell-depleting anti-CD20 therapies in MS; the first of these antibodies depleting immature and mature B cells trialed successfully in relapsing-remitting MS (RRMS) was rituximab (2, 3), which led to its extended off-label use. Ocrelizumab, its further humanized successor, has recently been approved for RRMS as well as for primary progressive MS (PPMS) (4, 5) based on its ability to drastically reduce the relapse rate in RRMS and to weaken development of disability in PPMS.

B cells have three main functions in the immune response in MS: They function as 1) antigen-presenting cells (APCs) (6, 7), 2) immunomodulators through cytokine secretion (8), and 3) precursor cells for antibody-producing plasma cells (9). The intrathecal presence of antibody-producing plasma cells (10) and of B cell aggregates in the meninges of MS patients (11, 12) further support an important role of B cells in the pathogenesis of MS. In the blood, different B cell subpopulations, naive and memory B cells, are distinguished by their cytokine profile. Interleukin-10 (IL-10) is almost exclusively produced by naive B cells; tumor necrosis factor-α (TNF-α) and lymphotoxin (LT) are largely produced by memory B cells (8). MS patients’ B cells show an increased secretion of proinflammatory cytokines IL-6 and TNF-α and a decreased secretion of the antiinflammatory cytokine IL-10 compared to healthy controls (13).

In MS, the B cell phenotype before and at/after repletion following anti-CD20 therapy and the influence of this depletion on the remaining immune populations (T and myeloid cells) is not completely elucidated. This is an important challenge for B cell-depleting therapies in MS as it may help to determine whether patients must be continuously depleted of B cells to maintain the clinical benefit; furthermore, such analysis may elucidate to what extent it may be necessary to develop a long-term therapeutic strategy, i.e., a subsequent therapy after anti-CD20 treatment cessation. To approach these important questions, the phenotype and function of B, T, and myeloid cells in MS patients treated with the anti-CD20 antibody rituximab were studied before and over a 24-mo period post treatment.

## Results

### B Cell Counts and Phenotype before Depletion.

**Fig. 1.**
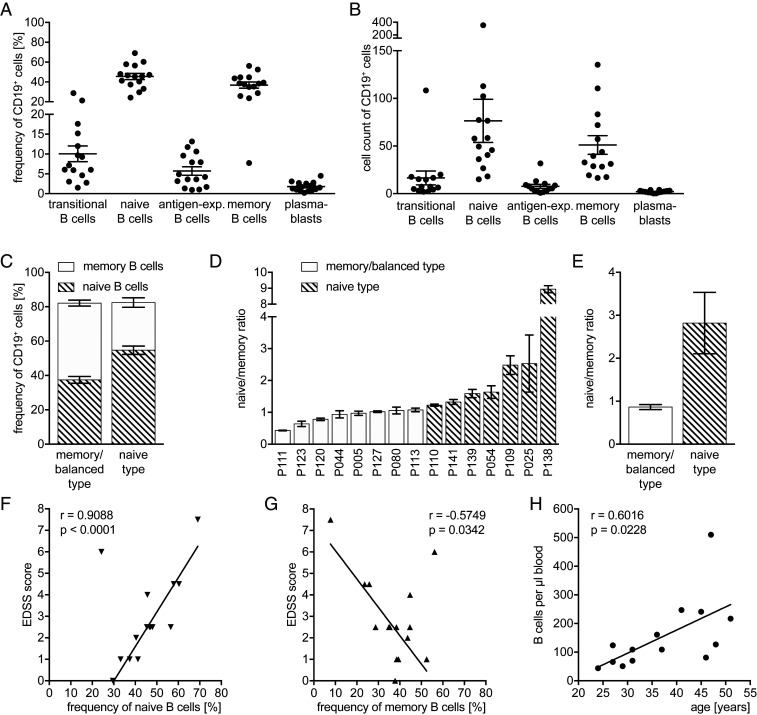
Individual B cell phenotype before therapy administration. Peripheral blood mononuclear cells were isolated from 15 MS patients before anti-CD20 antibody treatment was initiated (filled shapes, before depletion; *n* = 15 samples). Depicted are dot plots showing the mean ± SEM. Frequencies (*A*) and cell counts (*B*) of transitional (CD24^high^ CD38^high^), mature naive (CD27^−^ CD38^+^), antigen-experienced (=antigen-exp.; CD27^+^ CD38^+^), and memory (CD27^var^ CD38^−^) B cells as well as plasmablasts (CD20^−^ CD27^+^ CD38^+^) pre-gated on CD19^+^ B cells; frequency of transitional B cells and plasmablasts was gated within the mature naive respectively antigen-experienced B cells and was calculated to the B cell population. Second, frequency of transitional B cell and of plasmablasts was subtracted from mature naive respectively antigen-experienced B cells to receive the negative population. (*C*) Frequency of naive and memory B cells of all patients divided into two groups based on the naive/memory ratio. (*D*) Naive/memory ratio of every single patient in an increasing order and (*E*) mean naive/memory ratio of all patients, both (*E* and *F*) divided into two groups based on the naive/memory ratio (in memory/balanced type, ratio ≤ 1; in balanced type, the difference between the frequencies of naive and memory B cells must not exceed 5%; in naive type, ratio > 1). (*F*) Correlation between frequency of naive B cells before and EDSS score before treatment initiation (linear regression, Spearman *r*; when *n* = 15: *r* = 0.5938, *P* = 0.0216). (*G*) Correlation between frequency of memory B cells before and EDSS score before treatment initiation (linear regression, Spearman *r*; when *n* = 15: *r* = −0.3234, *P* = 0.2385). Correlation between B cell counts before first anti-CD20 antibody administration with age (*H*; *n* = 14); linear regression, Pearson *r*.

We phenotyped peripheral blood B cells in all patients before treatment initiation. As indicated in Fig. 1 *A* and *B*, the B cell pool of all 15 patients contained mainly naive (45.5 ± 3.1%; mean ± SEM) and memory B cells (36.8 ± 3.1%), yet their relative frequency was interindividually very heterogenous (Fig. 1 *C* and *D*). In order to investigate whether the individual phenotype correlates with disease activity or other clinical parameters, patients were stratified according to their main B cell population. The ratio of naive/memory B cells was used for this approach (Fig. 1 *D* and *E*; in memory/balanced type, ratio ≤ 1; in balanced type, the difference between the frequencies of naive and memory B cells must not exceed 5%; in naive type, ratio > 1). Interestingly, the patients’ expanded disability status scale (EDSS) score positively correlated with the frequency of naive (Fig. 1*F*), and negatively with that of memory B cells (Fig. 1*G*). There was no correlation of those parameters with age or disease duration (data not shown). Furthermore, we found a positive correlation between B cell counts and patients’ age, which was driven by the female population (Fig. 1*H*). A correlation between B cell counts and disease duration was not found (data not shown). Despite various pretreatments with different disease-modifying therapies and individual patients with a short washout period, the baseline results showed only minor variations in B cell counts and phenotype.

### B Cells Reappear with a Less Mature, yet Highly Activated Phenotype.

**Fig. 2.**
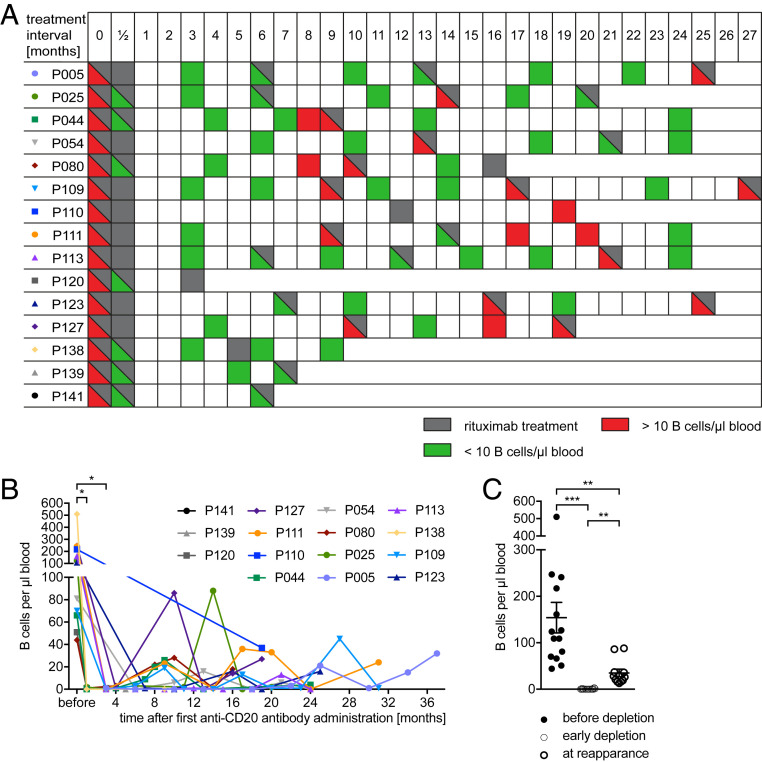
Individual anti-CD20 antibody treatment intervals and counts. Blood samples of MS patients (*n* = 15) were collected before anti-CD20 antibody treatment was initiated and at several time points thereafter. (*A*) Schematic illustration of the patients’ individual anti-CD20 antibody treatment intervals (gray, anti-CD20 antibody treatment) and blood sampling for the detection of B cells. Total B cells numbers were determined by a standard clinic’s blood evaluation and are shown schematically as follows: green, <10 B cells/µL blood; red, >10 B cells/µL blood. (*B*) Absolute B cell counts in whole blood determined by a standard clinic’s blood evaluation before anti-CD20 antibody treatment was initiated (*P < 0.05; Wilcoxon matched-pairs signed rank test). (*C*) B cell counts in the samples tested before anti-CD20 antibody treatment was initiated (filled shapes, before depletion; *n* = 14 samples), after 1 to 5 mo (=early depletion; *n* = 14), and after 8 to 24 mo (open shapes, at reappearance; *n* = 10 samples; **P < 0.005; ***P < 0.001; Wilcoxon matched-pairs signed rank test).

**Fig. 3.**
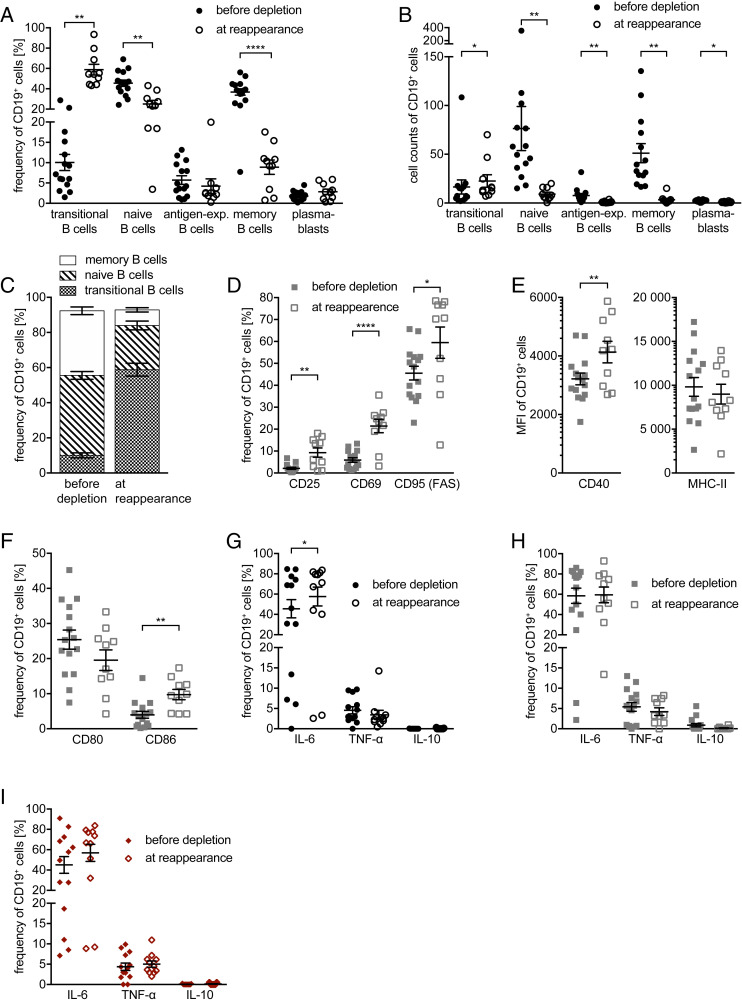
B cell maturation and phenotype before treatment initiation and at their reappearance. Peripheral blood mononuclear cells were isolated from 15 MS patients before anti-CD20 antibody treatment was initiated (filled shapes, before depletion; *n* = 15 samples) and after 8 to 24 mo (open shapes, at reappearance; *n* = 10 samples). Depicted are dot plots showing the mean ± SEM. Frequencies (*A*) and cell counts (*B*) of transitional (CD24^high^ CD38^high^), mature naive (CD27^−^ CD38^+^), antigen-experienced (=antigen-exp.; CD27^+^ CD38^+^), and memory (CD27^var^ CD38^−^) B cells as well as plasmablasts (CD20^−^ CD27^+^ CD38^+^) pre-gated on CD19^+^ B cells (*P < 0.05; **P < 0.005; ****P < 0.0001; Wilcoxon matched-pairs signed rank test/paired *t* test); frequency of transitional B cells and plasmablasts was gated within the mature naive respectively antigen-experienced B cells and was calculated to the B cell population. Second, frequency of transitional B cell and of plasmablasts was subtracted from mature naive respectively antigen-experienced B cells to receive the negative population. (*C*) Frequency of transitional, naïve, and memory B cells of all patients before depletion and at reappearance. Cells were cultured for 22 h in the presence of CpG (*D–F*; gray squares). Frequency (*D*) of CD19^+^ B cells expressing CD25, CD69, and CD95 (FAS) (*P < 0.05; **P < 0.005; ***P < 0.001; Wilcoxon matched-pairs signed rank test/paired *t* test). (*E*) B cells’ expression of CD40 and MHC class II (MHC-II) shown as mean fluorescence intensity (MFI) as well as (*F*) frequency of CD19^+^ B cells expressing CD80 and CD86 (**P < 0.005; Wilcoxon matched-pairs signed rank test/paired *t* test). Cells were cultured for 22 h unstimulated (*G*), in the presence of CpG (*H*), and 20 h unstimulated followed by 2 h in the presence of LPS (*I*), all followed by 4 additional hours in the presence of ionomycin, PMA, and GolgiPlug. Flow cytometry analysis of frequency of CD19^+^ B cells expressing tumor necrosis factor-α (TNF-α), interleukin-6 (IL-6), and IL-10 (*P < 0.05; Wilcoxon matched-pairs signed rank test).

In all of our 15 patients, anti-CD20 treatment was effective. During depletion, only one patient had a relapse (6 mo after treatment initiation in the absence of a new gadolinium enhancing MRI lesion) and another experienced a worsening of the EDSS 19 mo after treatment initiation. Regarding MRI activity, two patients had new MRI activity, both in the first 6 mo after induction of rituximab therapy. One patient had seven new T2 lesions in cranial MRI; the other had one new gadolinium enhancing lesion in spinal MRI. Both MRI activities occurred in complete B cell absence. Conversely, none of the patients with reemergence of B cells in the blood showed MRI activity. Accordingly, no association between B cell repopulation and MRI activity can be deducted from our cohort (Table 1; for detailed patient treatment regimens, see Fig. 2*A*). One primary goal of our study was to analyze the returning B cell phenotype. Phenotypically, B cells reappeared very homogeneously in all patients analyzed, although B cell repletion kinetics varied interindividually and overall reappearance occurred in different magnitudes (Fig. 2 *B* and *C*). Specifically, reoccurring B cells were highly enriched in transitional B cells (58.85 ± 5.281%) and strongly diminished in memory B cells (8.9 ± 1.768%). Furthermore, the frequency of naive B cells was reduced from 45.5% to 25.1% ± 3.5%, while antigen-experienced B cells and plasmablasts remained unchanged. Overall, at an early time point in the repletion phase, B cells had a dominant transitional/naive phenotype (Fig. 3 *A*–*C*).

**Table 1.** Characteristics of the patient cohort

Characteristic	Study cohort
Number of patients (samples before/depletion/early repletion/late repletion)	15 (15/12/10/10)
Sex, W/M	8/7
EDSS score at start of the study, median, IQR/range	2.5 ± 3.5
Age at start of study, y, mean ± SD	35.73 ± 8.91
Time since MS diagnosis, y, mean ± SD	10.76 ± 6.31
Observation time after initiation of anti-CD20 treatment, y, mean ± SD	2.68 ± 0.658
Last treatment before rituximab (cases)	
Dimethylfumarate	2
Fingolimod	6
Glatiramer acetate	1
Natalizumab	2
Azathioprine	1
None (i.e., treatment naive)	3
Washout periods, mo, mean ± SD	5.42 ± 4.56
Prior treatments (cases)	
Dimethylfumarate	2
Fingolimod	9
Glatiramer acetate	4
IFNβ-1a	6
IFNβ-1b	2
Natalizumab	6
Azathioprine	1
Cortisone (in last 6 mo before first anti-CD20 treatment)	4
None (i.e., treatment naive)	3
Patients with relapses after therapy initiation	1, yet with no MRI activity
Patients with EDSS deterioration after therapy initiation	1, yet after 19 mo since treatment initiation, without new MRI activity

Next, we aimed to compare the activation state and antigen-presenting potential of reappearing B cells with the preexisting phenotype before anti-CD20 treatment. B cells before depletion showed a relatively uniform expression of proliferation and activation markers, with a low CD25 and CD69 expression, and a higher expression of CD95 (FAS). Strikingly, the repopulating B cells consistently showed a significantly higher expression of these markers, indicating a more activated status (Fig. 3*D*). Along the same lines, the reappearing B cell pool showed an enhanced expression of CD40 and CD86, whereas MHC class II and CD80 remained unchanged (Fig. 3 *E* and *F*). In conjunction, these findings point toward repopulating B cells being strongly activated and having a higher costimulatory potential.

### Reappearing B Cells Secrete More IL-6.

Reappearing B cells showed an increase in basal IL-6 secretion compared to B cells before anti-CD20 treatment, while the frequency of IL-10– and TNF-α–secreting B cells remained unchanged. Of interest, when comparing B cells in a stimulated and in an unstimulated state, B cells before anti-CD20 treatment showed an up-regulation of their IL-6 secretion, while reappearing B cells already in the nonstimulated setting showed an elevated IL-6 production (Fig. 3*G*), which could not further be enhanced by CpG or LPS stimulation (Fig. 3 *H* and *I*). These data support the assumption that reoccurring B cells have a reinforced proinflammatory capacity.

### CD4^+^ and CD8^+^ T Cells React Alike to Anti-CD20 Depletion with a Relative Loss in Effector Function.

**Fig. 4.**
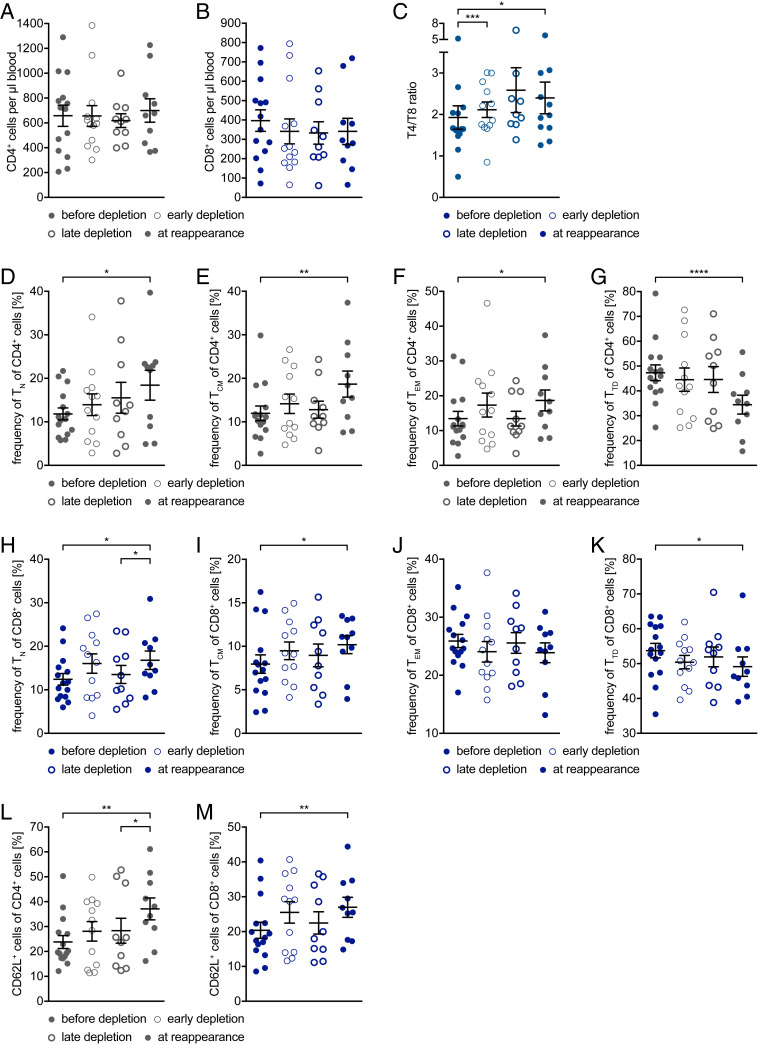
T cell maturation phenotype before treatment initiation and during the depletion and repletion phases. Peripheral blood mononuclear cells were isolated from 15 MS patients before anti-CD20 antibody treatment was initiated (filled circles, before depletion; *n* = 15 samples), after 1 to 5 mo (thin circles, early depletion; *n* = 12 samples), after 6 to 8 mo (thick circles, late depletion *n* = 10 samples), and after 8 to 24 mo (filled circles, at reappearance = at B cell reappearance; *n* = 10 samples). Depicted are dot plots showing the mean ± SEM. (*A*) Cell count of CD4^+^ cells (gray circles). (*B*) Cell count of CD8^+^ cells (blue circles). (*C*) Ratio between cell count of CD4^+^ cells and of CD8^+^ cells in whole blood (gray blue circles; *P < 0.05; ***P < 0.001; Wilcoxon matched-pairs signed rank test; α value was corrected with the Bonferroni–Holm method). Frequency of naive (TN, CD62L^+^ CD45RO^−^; *D*), central memory (TCM, CD62L^+^ CD45RO^+^; *E*), effector memory (TEM, CD62L^−^ CD45RO^+^; *F*), and terminally differentiated (TTD, CD62L^−^ CD45RO^−^; *G*) cells gated on CD4^+^ cells (*P < 0.05; **P < 0.005; ****P < 0.0001; Wilcoxon matched-pairs signed rank test/paired *t* test; α value was corrected with the Bonferroni–Holm method). Frequency of naive (TN, CD62L^+^ CD45RO^−^; *H*), central memory (TCM, CD62L^+^ CD45RO^+^; *I*), effector memory (TEM, CD62L^−^ CD45RO^+^; *J*), and terminally differentiated (TTD, CD62L^−^ CD45RO^−^; *K*) cells gated on CD8^+^ cells (*P < 0.05; paired *t* test; α value was corrected with the Bonferroni–Holm method). Frequency of (*L*) CD62L^+^ CD4^+^ cells and of (*M*) CD62L^+^ CD8^+^ cells (*P < 0.05; **P < 0.005; Wilcoxon matched-pairs signed rank test/paired *t* test; α value was corrected with the Bonferroni–Holm method).

We further analyzed the impact on the frequency, phenotype, and function of T cells. For this purpose, we compared four different time points, before depletion, an early time point thereafter, late-stage depletion, and B cell reappearance (8 to 24 mo after treatment). The total number of CD4^+^ and CD8^+^ T cells did not change significantly upon anti-CD20–mediated cell depletion (Fig. 4 *A* and *B*), nor did their state of differentiation (Fig. 4 *D*–*K*; before depletion vs. early depletion; after 1 to 5 mo). CD4^+^ T cell dominance increased, which is reflected in the significant increase in the ratio between CD4^+^ and CD8^+^ T cells (Fig. 4*C*). However, when comparing the CD4^+^ T cell differentiation state before therapy with the time point at B cell reoccurrence, we observed an increase in naive, central memory, and effector memory T cell frequencies (Fig. 4 *D*–*F*), while the frequency of terminally differentiated T cells (Fig. 4*G*) was strongly decreased. Although less pronounced, similar findings could be observed for CD8^+^ T cells, except for the frequency of effector memory cells, which remained unaltered for all time points investigated (Fig. 4 *H*–*K*). In addition to the changes in T cell maturation, we observed a strong increase in the expression of the adhesion molecule CD62L by both CD4^+^ and CD8^+^ T cells (Fig. 4 *L* and *M*; before depletion vs. at reappearance; after 8 to 24 mo), indicating a higher capacity of these cells to home to secondary lymphoid organs.

### Preexisting B Cell Phenotype Determines T Cell Differentiation following Depletion and Repletion.

**Fig. 5.**
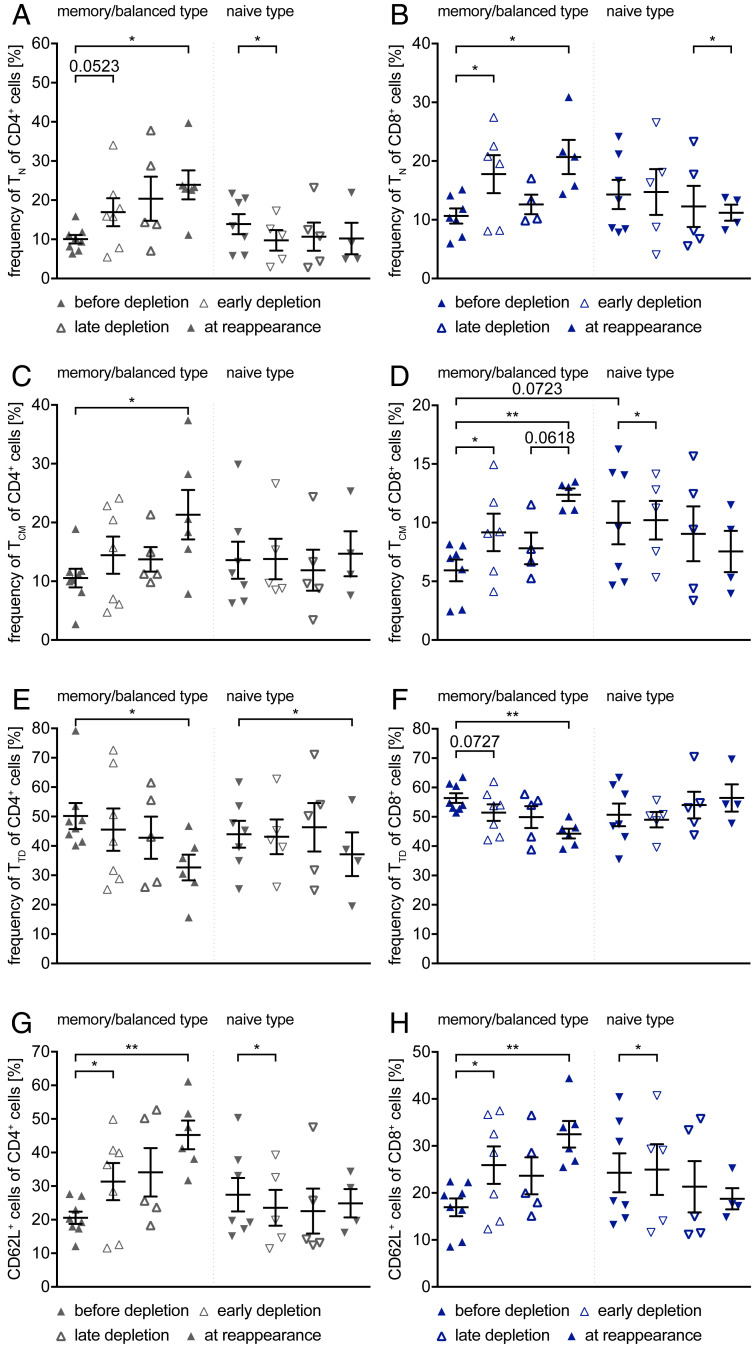
Differences in T cell maturation phenotype and CD62L expression between memory/balanced and naive type. Peripheral blood mononuclear cells were isolated from 15 MS patients before anti-CD20 antibody treatment was initiated (filled triangles, before depletion; *n* = 8/7 samples), after 1 to 5 mo (thin triangles, early depletion; *n* = 7/5 samples), after 6 to 8 mo (thick triangles, late depletion; *n* = 5/5 samples), and after 8 to 24 mo (filled triangles, at reappearance = at B cell reappearance; *n* = 6/4 samples). The patients were classified as memory/balanced type or naive type as described in Fig. 1. Depicted are dot plots showing the mean ± SEM. Frequency of naive cells (T_N_, CD62L^+^ CD45RO^−^) gated on CD4^+^ cells (*A*) and on CD8^+^ cells (*B*), central memory cells (T_CM_, CD62L^+^ CD45RO^+^) gated on CD4^+^ cells (*C*) and on CD8^+^ cells (*D*), and terminally differentiated cells (T_TD_, CD62L^−^ CD45RO^−^; C) gated on CD4^+^ cells (*E*) and on CD8^+^ cells (*F*), as well as frequency of CD62L^+^ CD4^+^ T cells (*G*) and CD8^+^ T cells (*H*) (*P < 0.05; **P < 0.005; Wilcoxon matched-pairs signed rank test/paired *t* test; α value was corrected with the Bonferroni–Holm method; difference between the two groups [D]: unpaired *t* test).

Next, we aimed at examining whether the preexisting B cell phenotype may correlate with differential changes in T cell maturation. For this purpose, we divided our cohort as mentioned earlier by the relative dominance of naive versus memory B cells in the pre–anti-CD20 blood sample (Fig. 1 *C*–*E*). When comparing the T cell differentiation before treatment initiation between these two groups (Fig. 5), we detected a greater value scattering from patients with a predominantly naive B cell phenotype before depletion in both T cell subpopulations (CD4^+^ *P* = 0.0476; Mann–Whitney *U* test; CD8^+^ *P* = 0.0343; unpaired *t* test). Furthermore, patients with a memory/balanced B cell type revealed in the long term (before depletion vs. at reappearance; after 8 to 24 mo) increased frequencies of naive and central memory CD4^+^ and CD8^+^ T cells, along with an increase in CD62L expression, and a complementary decrease in frequency of terminally differentiated T cells. In contrast, patients with a naive B cell phenotype showed, with exception of a minor decrease in terminally differentiated, no changes in CD4^+^ T cell maturation, with minimal changes in CD62L expression. Patients with a naive B cell type showed the following changes in CD8^+^ T cell maturation: decrease in naive and central memory T cells with a complementary increase in terminally differentiated T cells, with minimal changes in CD62L expression.

### CD14^+^ Myeloid Cells Show Transient Changes upon Anti-CD20 Antibody Treatment.

**Fig. 6.**
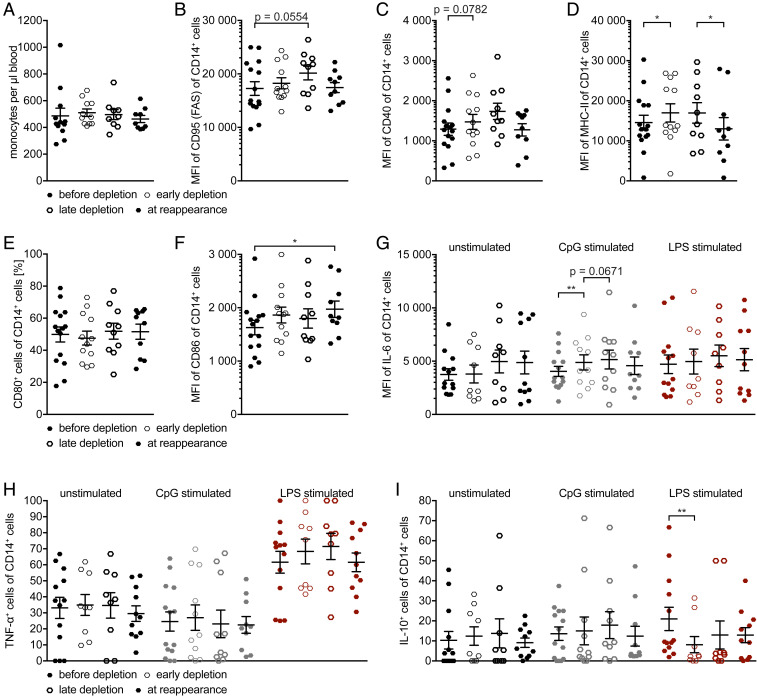
Myeloid cells phenotype and global cytokine analysis before treatment initiation and during the depletion and repletion phases. Peripheral blood mononuclear cells were isolated from 15 MS patients before anti-CD20 antibody treatment was initiated (filled hexagons, before depletion; *n* = 15 samples), after 1 to 5 mo (thin hexagons, early depletion; *n* = 12 samples), after 6 to 8 mo (thick hexagons, late depletion; *n* = 10 samples), and after 8 to 24 mo (filled hexagons, at reappearance, at B cell reappearance; *n* = 10 samples). Depicted are dot plots showing the mean ± SEM. (*A*) Cell counts of monocytes (black hexagons). Myeloid cells’ expression of CD95 (FAS; *B*), CD40 (*C*), and MHC class II (MHC-II; *D*) shown as mean fluorescence intensity (MFI) (*P < 0.05; Wilcoxon matched-pairs signed rank test/paired *t* test; α value was corrected with the Bonferroni–Holm method) as well as (*E*) frequency of CD14^+^ myeloid cells expressing CD80 and (*F*) myeloid cells’ CD86 expression shown as mean fluorescence intensity (MFI) (*P < 0.05; paired *t* test). Cells were cultured for 22 h unstimulated (*G*–*I*; black hexagons), 22 h in the presence of CpG (gray hexagons), and for 20 h unstimulated followed by 2 h in the presence of LPS (red hexagons), all followed by four additional 2 h in the presence of ionomycin, PMA, and GolgiPlug. Flow cytometry analysis of (*G*) CD14^+^ myeloid cells’ expression of interleukin-6 (IL-6) shown as mean fluorescence intensity (MFI) (**P < 0.005; Wilcoxon matched-pairs signed rank test/paired *t* test; α value was corrected with the Bonferroni–Holm method), frequency of CD14^+^ myeloid cells’ expressing (*H*) tumor necrosis factor-α (TNF-α) and (*I*) IL-10 (**P < 0.005; paired *t* test).

The total number of monocytes was not altered upon anti-CD20–mediated cell depletion (Fig. 6*A*). Comparing the phenotype of myeloid cells before B cell depletion and during B cell absence (early depletion; after 1 to 5 mo), the following changes were found: a nonsignificant increase in CD40 expression, a significant increase in MHC II expression, and significant changes in cytokine production, i.e., increase in IL-6 and a decrease in IL-10 production. Over the course of B cell reappearance (at reappearance; after 8 to 24 mo), normalization of a transiently enhanced CD95 (FAS), CD40, and MHC II expression and a continuously increasing CD86 expression were observed. IL-6 and IL-10 normalized, albeit not to original level. TNF-α production remained unchanged (Fig. 6 *B*–*H*). When looking in greater detail on the unstimulated IL-6 production (Fig. 6*G*), one could see that the myeloid cell reaction spreads; some show over time an increased IL-6 production and some show a decreased one. Altogether, these observations suggest that nonselective, anti-CD20–mediated pan B cell depletion results in cessation of B cell antiinflammatory regulation of myeloid cells, and that myeloid cells overtake the B cells’ APC function. As the majority of these changes are transient (with the exception of CD86), one could see the return of B cell antiinflammatory regulation of myeloid cells.

## Discussion

We here aimed at characterizing the immunological consequences of anti-CD20–mediated B cell depletion, with a particular focus on how B cells reappear after their removal and how the absence and reoccurrence of B cells impact frequency, differentiation, and activity of T and myeloid cells. These investigations aimed at unraveling how this extremely effective treatment option will be used most wisely in the context of a long-term effective MS therapy regimen. Being able to anticipate in what status B cells return after anti-CD20 treatment is of particular relevance, since continuous B cell depletion intrinsically harbors the risk of developing humoral deficiencies over time, which may require treatment cessation and even immunoglobulin substitution. In addition, anti-CD20 treatment may directly or indirectly affect T and myeloid cells with consequences for follow-up treatment regimens.

In all patients scheduled to receive anti-CD20, we first characterized in detail the target population of peripheral B cells. Of interest, we noted that MS patients showed a positive correlation between B cell counts and age. B cell counts in peripheral blood of healthy individuals increase until the age of ∼26 y of age, and then stabilize until the age of 50 (14, 15).

When looking at the distribution between the two genders, this correlation was driven primarily by the female population included. This group showed generally a lower B cell count, yet still within the norm. This is surprising, since in general the B cell compartment does not show a significant gender specific distribution (15). We thus hypothesize that the observed association relates to MS or autoimmunity in general. It is known that certain B cells subpopulations are more frequent in elderly women with autoimmune disorders than in young women, or men of any age. At this point, we can only speculate that the observed increase of the B cell counts with increasing age in women relates to an expansion of the reported B cell population (16).

All MS patients showed two main B cell phenotypes before treatment, naive and memory B cells. Individual patients yet differed in the relative composition of these differential B cell phenotypes. We found in all but one patient a strong correlation between the naive and memory B cell frequencies and the EDSS score (which should be interpreted very cautiously in light of the small number of patients). Furthermore, we detected that patients with a predominantly memory/balanced B cell phenotype had a lower EDSS score when compared to patients with predominantly naive peripheral B cells. This comes as a surprise, as memory B cells are thought to have a more proinflammatory phenotype (8), and their increase worsens the clinical activity of MS (17).

In view of these variations within our cohort, B cells repleted in a surprisingly homogenous manner after cessation of anti-CD20 treatment. Overall, repleting B cells were less mature, mostly transitional and also naive. Transitional B cells are the least mature B cells capable of migrating from the bone marrow to the periphery (18), suggestive of a de novo repletion from the bone marrow. The other important observation was that repleting B cells showed a more activated phenotype when compared to their phenotype before depletion. This is seen in the elevated expression of activation markers (among others, CD25), in the higher expression of costimulatory molecules, and in the cytokine profile, showing a relative decrease in antiinflammatory cytokine secretion (IL-10) and an increase in proinflammatory cytokine secretion (IL-6). CD25 is mostly expressed by memory B cells (19), which contain a higher frequency of activated, proliferating B cells with enhanced antigen presentation capacities (20). In context with our phenotypical analysis above, we do not believe that the higher expression of CD25 is due to an elevated frequency of memory B cells in the repleting pool, but reflective of the fact that naive, regrowing B cells are being activated in the process of repopulation. This general observation confirms our earlier experimental study showing that in the context of peripheral activation, B cells reappear with an enhanced proinflammatory molecular arsenal (21). Jointly, these data point to a shift toward a less differentiated state, yet with enhanced proliferation, activation, and costimulatory capacities. Of note, similar results were also found by analyzing the pool of repopulating B cells after CD20 depletion with rituximab in other autoimmune diseases (22).

CD4^+^ and CD8^+^ T cells reacted alike to anti-CD20 depletion and showed a relative loss in effector function (13), and a decrease in terminally differentiated T cells (the CD4^+^ larger than CD8^+^), an increase of CD4^+^ respectively constant CD8^+^ effector memory T cells. Of note, both T cell populations have the highest immediate effector functions. In general, these changes in the T cell compartment may occur as an indirect consequence of removing B cells. Alternatively, this may be a direct effect, as a proportion of highly differentiated, cytokine-producing effector CD4 and CD8 T cells is nowadays known to express CD20 (23, 24), which may lead to their extinction upon anti-CD20 treatment (25, 26). To distinguish between these two possibilities will be crucial in the future yet exceeded the scope of this pilot study. Nevertheless, as a first approach in this direction, we correlated the preexisting B cell phenotype with the effect of anti-CD20 treatment within the T cell compartment. Of note, the patients with memory/balanced B cell phenotype showed relatively extended differences in the T cell phenotype upon B cell depletion, suggesting a loss of T cell stimulation by differentiated B cells (27). On the other hand, the patients with a naive B cell phenotype showed partially opposite changes in the T cell phenotype. Within the compartment of myeloid cells, pan B cell depletion was associated with an up-regulated activity of myeloid cells in the blood. Of note and in contrast to T cells, these changes were not determined by the preexisting B cell phenotype. Most likely, these alterations, which confirm our earlier observations in mice (21) and men (28) represent the concomitant loss of B cell regulatory properties (29–31), which likely occurs upon nonselective depletion of CD20-positive B cells.

In conclusion, the process of therapeutically removing CD20-positive cells in patients with MS and related diseases is immunologically more complex than previously thought. Besides unselectively removing B cells, anti-CD20 treatment is associated with profound changes in the T cell compartment as well as with detectable alterations in the phenotype and function of myeloid cells. Furthermore, upon cessation of treatment, B cells reappear in a relatively immature status yet with a substantial incline in the expression of activation markers and in the release of proinflammatory cytokines. This primary observation indicates that regeneration of B cells after their anti-CD20–mediated removal is an active process rather than a simple, physiological regrowth of B cells with the same properties as before. Whether our immunological observations lead to clinical reactivation of the disease remains open. A large observational study did not find evidence of accentuated disease reappearance in a large patient cohort (32), while it is possible that individual patients may react differently. Regarding a possible transition of RRMS patients to secondary progressive MS, it was shown that rituximab may not necessarily prevent it (33). This may indicate that anti-CD20 is extremely powerful in prevention of relapses and de novo lesion formation, while its effect on secondary progression is questionable. All analyses provided here are generated from patients treated with rituximab, as reoccurrence of B cells is extremely rare with ocrelizumab due to the usually constant and relatively short treatment interval. Nevertheless, as rituximab and other anti-CD20 deplete the identical population of B cells, it is reasonable to assume that ocrelizumab and ofatumumab might have similar effects. While it remains to be determined whether this change in our conception of anti-CD20 is of direct clinical impact, it may be instructive in the development of sustainable, possibly sequential, B cell-directed treatment strategies beyond continuous anti-CD20 therapies.

## Materials and Methods

### Patients.

Fifteen RRMS patients were enrolled at the University Medical Center Göttingen, Germany, after written informed consent; this study was approved by the ethics committee of the University Medical Center Göttingen (#19/09/10 and #03/04/14). Since rituximab is an off-label treatment for MS, the number of includable patients at our center was limited to 15. Eight females and seven males were included; their mean age was 35.7 y, mean disease duration was 10.7 y, and median EDSS score was 2.5. For detailed patient characteristics, see Table 1. All patients received rituximab at a dose of 1,000 mg on days 1 and 15, followed by 1,000 mg every 3 to 15 mo. For detailed patient treatment regimens, see Fig. 2*A*. Blood samples were collected during routine clinical assessment before anti-CD20 antibody treatment and at several time points thereafter. Immune cell counts were determined from whole blood in the hospital’s routine laboratory. Detailed descriptions of study materials and methods are provided in *SI Appendix*, *SI Materials and Methods*.

## Supplementary Material

Supplementary File

## Data Availability

All study data are included in the article and *SI Appendix*.
