# Supplementary material for: B cells reappear less mature and more activated after their anti-CD20–mediated depletion in multiple sclerosis
Source: Proc Natl Acad Sci U S A. 2020 Sep 30;117(41):25690–9. doi: 10.1073/pnas.2012249117 (PMC7568262; doi:10.1073/pnas.2012249117)
Supplement: Supplementary File [file pnas.2012249117.sapp.pdf]

PNAS
www.pnas.org

Supplementary Information for

B cells reappear less mature and more activated after their anti-CD20 mediated depletion in multiple sclerosis

Nitzan Nissimov[1], Zivar Hajiyeva MD[2], Sebastian Torke PhD[1], Katja Grondey[1],
Wolfgang Brück MD[1], Silke Häusser-Kinzel PhD[1], Martin S. Weber MD[1,2]

[1]Institute of Neuropathology, University Medical Center, Göttingen, Germany
[2]Department of Neurology, University Medical Center, Göttingen, Germany

Corresponding author: Martin S. Weber, MD
Corresponding author's address: Department of Neuropathology
Department of Neurology
University Medical Center, Georg August University
Robert-Koch-Str. 40, 37099 Göttingen, Germany
Corresponding author's phone and fax: Phone +49-551 39-7706, Fax +49-551-39-10800
Corresponding author's e-mail address: martin.weber@med.uni-goettingen.de

This PDF file includes:

- Supplementary text
- Figures S1
- Tables S1 to S2

www.pnas.org/cgi/doi/10.1073/pnas.2012249117

Supplementary Information Text

SI Materials and Methods

PBMCs preparation and stimulation

PBMCs were isolated by Ficoll density gradient (for detailed list of manufacturers see **Table S1**) and cryopreserved at -80°C. For analysis, cells were thawed and plated. For the analysis of activation marker and co-stimulatory molecules, PBMCs were stimulated with 2µg/ml CpG for 22 hours at 37°C and 5% CO_2. For cytokine assessment, PBMC were cultured unstimulated for 22 hours, in the presence of 1µg/ml CpG for 22 hours or 20 hours unstimulated followed by 2 hours with 500pg/ml LPS. All pre-stimulation regimes were then incubated with 500ng/ml ionomycin, 20ng/ml phorbol 12-myristate 13-acetate and Golgi Plug for 4 hours.

Flow cytometry

Cells were pre-incubated with Fc receptor blocking solution and stained with viability dye (Zombie Dye, 1:500) for live/dead cell discrimination. For analysis of intracellular cytokines, cells were permeabilized by adding fixation/permeabilization solution. All panels and FACS antibodies used are listed in **Table S2**. Samples were analyzed on a LSRII Fortessa. All samples of the same patient were stained and analyzed in batch in order to limit the inter-assay variability. To ensure cell viability the live/dead cell discrimination was tested per panel: Panel 1 (95.88%± 1.64%; mean ± SD), Panel 2 (93.27% ± 3.58%), Panel 3 (92.65% ± 3.53%), Panel 4 (55.76% ± 14.05%), Panel 5 unstimulated (56.13% ± 15.17%), with CpG stimulated (63.94% ± 17.32%), with LPS stimulated (60.34% ± 13.60%).

Statistical analysis

Patients' data were tested for Gauss distribution with the Shapiro-Wilk normality test and the Kolmogorov-Smirnov normality test. For two longitudinal parametric samples paired t-test was used, for non-parametric Wilcoxon matched-pairs signed rank test. α-value was corrected with the Bonferroni-Holm method. For possible correlations linear regression was used, for interval scale Spearman r, for rational scale Pearson r. A p-value < 0.05 was considered statistically significant. Patients' data were classified into 4 time points: (1) before treatment initiation (= before depletion) (2) after one to five months (= early depletion), (3) after six to eight months (= late depletion; both early and late depletion time points are at B cell absence and only differ through time) and (4) after eight to 24 months (= at reappearance; = at B cell reappearance). All sample timings are from baseline; i.e. from the first treatment administration. Since patient sampling occurred individually in the clinical routine, not all samples from each patient were obtained at each time point, and therefore sample counts change between the different sampling time points, and a multi-comparison test could not be performed.

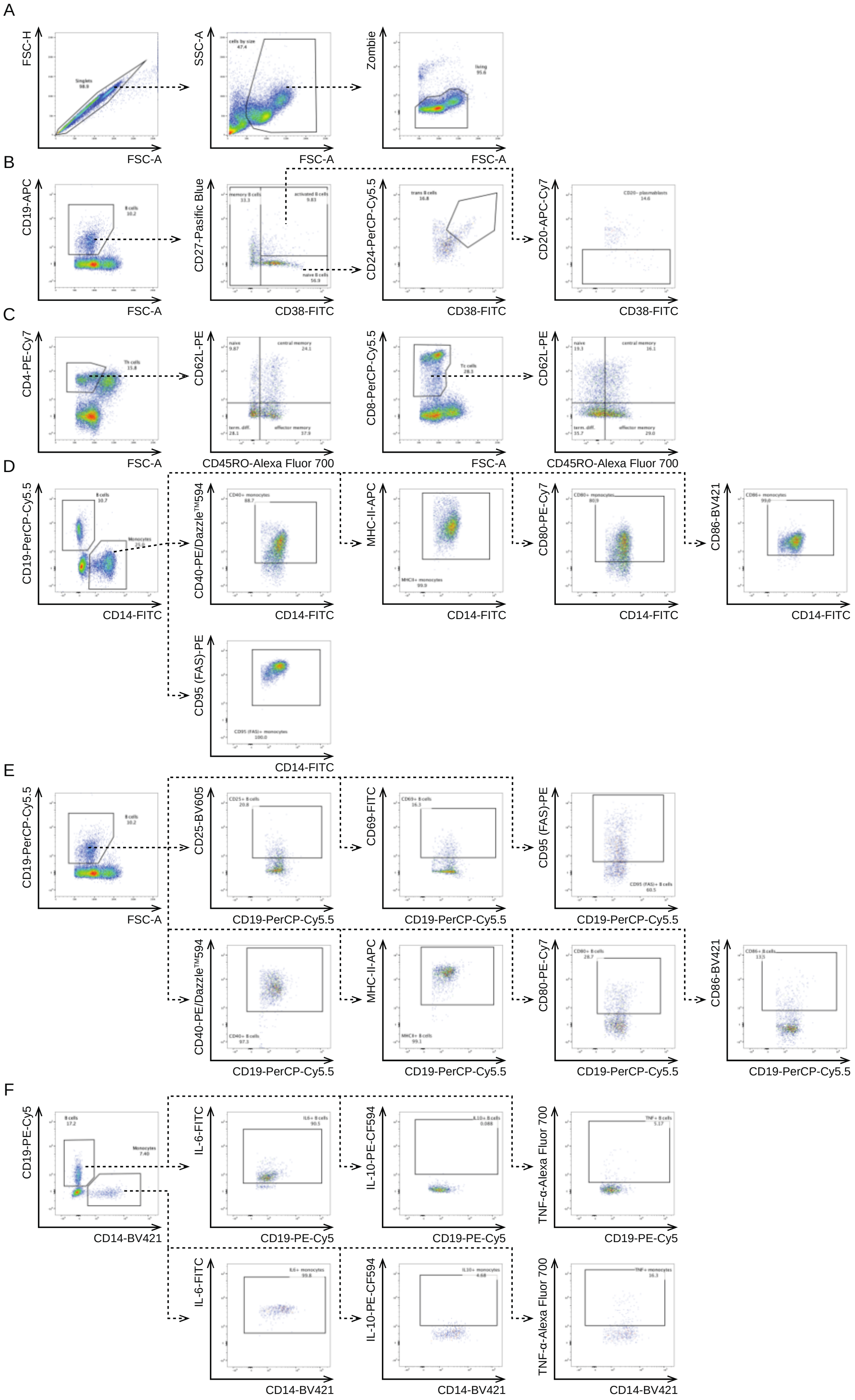
A
FSC-H
FSC-A
SSC-A
FSC-A
Zombie
FSC-A
B
CD19-APC
FSC-A
CD27-Pasific Blue
CD38-FITC
CD24-PerCP-Cy5.5
CD38-FITC
CD20-APC-Cy7
CD38-FITC
C
CD4-PE-Cy7
FSC-A
CD62L-PE
CD45RO-Alexa Fluor 700
CD8-PerCP-Cy5.5
FSC-A
CD62L-PE
CD45RO-Alexa Fluor 700
D
CD19-PerCP-Cy5.5
CD14-FITC
CD40-PE/Dazzle™594
CD14-FITC
MHC-II-APC
CD14-FITC
CD80-PE-Cy7
CD14-FITC
CD86-BV421
CD14-FITC
CD95 (FAS)-PE
CD14-FITC
E
CD19-PerCP-Cy5.5
FSC-A
CD25-BV605
CD19-PerCP-Cy5.5
CD69-FITC
CD19-PerCP-Cy5.5
CD95 (FAS)-PE
CD19-PerCP-Cy5.5
CD40-PE/Dazzle™594
CD19-PerCP-Cy5.5
MHC-II-APC
CD19-PerCP-Cy5.5
CD80-PE-Cy7
CD19-PerCP-Cy5.5
CD86-BV421
CD19-PerCP-Cy5.5
F
CD19-PE-Cy5
CD14-BV421
IL-6-FITC
CD19-PE-Cy5
IL-10-PE-CF594
CD19-PE-Cy5
TNF-α-Alexa Fluor 700
CD19-PE-Cy5
IL-6-FITC
CD14-BV421
IL-10-PE-CF594
CD14-BV421
TNF-α-Alexa Fluor 700
CD14-BV421

Fig. S1. Gating strategies for flow cytometry analysis. Peripheral blood mononuclear cells were isolated from 15 multiple sclerosis patients before anti-CD20 antibody treatment was initiated and at intervals thereafter. All gates were set based on the unstained sample (A) Pre-gating strategy. (B) Gating strategy for panel #1. (C) Gating strategy for panel #2. (D) Gating strategy for panel #3. (E) Gating strategy for panel #4. (F) Gating strategy for panel #5.

Table S1. List of materials

Product	Manufacturer
96 well plates, round bottom (U-well)	Sarstedt
BioColl separation solution	Biochrom
CpG oligodeoxynucleotides	Sigma Aldrich
Cytofix/CytopermTM	BD Biosciences
DMEM (Dulbecco's Modified Eagle's medium)	Sigma Aldrich
DMSO (dimethyl-sulfoxide)	Sigma Aldrich
EDTA (ethylene diamine tetraacetic acid disodium salt dihydrate)	Carl Roth
Ethanol 100%	Merck Millipore
Fc-BlockTM	BioLegend
FCS (fetal calf serum)	Sigma Aldrich
GolgiPlugTM	BD Biosciences
Ionomycin	Sigma Aldrich
L-glutamine	Sigma Aldrich
LPS (lipopolysaccharide)	Sigma Aldrich
PBS (phosphate buffered salt solution)	Sigma Aldrich
Perm/WashTM buffer, 10x	BD Biosciences
PMA (phorbol 12-myristrate 13 acetate)	Sigma Aldrich
RPMI-1640 (Roswell park memorial institute-1640)	Sigma Aldrich
Sodium pyruvate 100mM	Sigma Aldrich
TrypanBlue	Sigma Aldrich
β-mercaptoethanol	Sigma Aldrich

Table S2. List of flow cytometry panels and antibodies.

Panel	Antigen	Fluorochrome	Clone	Manufacturer
**#1 B cell subset**	CD19	APC	HIB19	BD Bioscience, NJ
	CD20	APC-Cy7	L27	BD Bioscience, NJ
	CD24	PerCp-Cy5.5	ML5	BioLegend, CA
	CD27	PacificBlue	O323	BioLegend, CA
	CD38	FITC	HIT2	BioLegend, CA
	Dead/Live	ZombieAquaTM		BioLegend, CA
**#2 T cell subset**	CD4	PE-Cy7	RPA-T4	BD Bioscience, NJ
	CD8	PerCP-Cy5.5	RPA-T8	BD Bioscience, NJ
	CD20	APC-Cy7	L27	BD Bioscience, NJ
	CD45RO	A700	UCHL1	BD Bioscience, NJ
	CD62L	PE	DREG-56	BD Bioscience, NJ
	Dead/Live	ZombieAquaTM		BioLegend, CA
**#3 APC activation**	CD14	FITC	M5E2	BD Bioscience, NJ
	CD19	PerCP-Cy5.5	HIB19	BioLegend, CA
	CD40	PE/DazzleTM594	5C3	BioLegend, CA
	CD80	PE-Cy7	L307.4	BD Bioscience, NJ
	CD86	BV421	2331 (FUN-1)	BD Bioscience, NJ
	CD95 (FAS)	PE	DX2	BioLegend, CA
	MHCII	APC	Tü36	BioLegend, CA
	Dead/Live	ZombieNIRTM		BioLegend, CA
**#4 B cell activation**	CD19	PerCP-Cy5.5	HIB19	BioLegend, CA
	CD25	BV605	BC96	BioLegend, CA
	CD40	PE/DazzleTM594	5C3	BioLegend, CA
	CD69	FITC	FN50	BioLegend, CA
	CD80	PE-Cy7	L307.4	BD Bioscience, NJ
	CD86	BV421	2331 (FUN-1)	BD Bioscience, NJ
	CD95 (FAS)	PE	DX2	BioLegend, CA
	MHCII	APC	Tü36	BioLegend, CA
	Dead/Live	ZombieNIRTM		BioLegend, CA
**#5 Cytokine secretion**	CD14	BV421	MφP9	BD Bioscience, NJ
	CD19	PE-Cy5	HIB19	BD Bioscience, NJ
	IL-6	FITC	MQ2-13A5	BD Bioscience, NJ
	IL-10	PE-CF594	JES3-19F1	BD Bioscience, NJ
	TNF-α	A700	MAb11	BD Bioscience, NJ
	Dead/Live	ZombieNIRTM		BioLegend, CA
